# Transanal endoscopic microsurgery: what indications in 2013?

**DOI:** 10.1093/gastro/got012

**Published:** 2013-04-05

**Authors:** Mario Morino, Marco E. Allaix

**Affiliations:** Digestive, Colorectal, Oncologic and Minimally Invasive Surgery, Department of Surgical Sciences, University of Turin, Italy

**Keywords:** transanal endoscopic microsurgery, full-thickness excision, rectal adenoma, early rectal cancer, chemoradiation, NOTES

## Abstract

Thanks to major advances in the field of surgical techniques and neoadjuvant chemoradiation therapy, along with more accurate pre-operative staging tools and the widespread introduction of population-based screening programs, treatment of rectal cancer has been evolving over the past few decades, moving towards a more tailored approach. This has brought a shift in the treatment algorithm of benign rectal lesions and selected early rectal cancers, for which today transanal endoscopic microsurgery (TEM) is accepted as an effective alternative to abdominal surgery.

In 2013, topics of controversy are the role of TEM in the treatment of more advanced rectal cancers, in cases of complete pathological response after chemoradiation therapy and the role of TEM as a platform for single-port surgery and NOTES. This article reviews the current indications for TEM and the future perspectives of this approach in the treatment of rectal tumors.

## INTRODUCTION

Transanal endoscopic microsurgery (TEM) is a minimally invasive technique that was conceived almost 30 years ago as an alternative to abdominal rectal resection and conventional transanal techniques, for the removal of large polyps localized in the rectum and not amenable to endoscopic resection [1]. While transanal local excision with retractors is associated with a significant incidence of local recurrence—in particular for tumors located in the proximal rectum [2, 3], TEM provides a transanal approach with low recurrence rates, thanks to an extremely precise dissection due to enhanced and stable visualization of the surgical field. In addition, the full-thickness *en bloc* excision allows accurate pathological evaluation of the specimen with precise staging of the disease.

Today, abdominal rectal resection, combined with total mesorectal excision (TME), is the ‘gold standard’ in the surgical treatment of rectal cancer. However, the postoperative course is burdened by significant mortality and morbidity [4–7]. Compared with abdominal surgery, TEM offers the advantage of combining a minimally invasive approach with evident benefits in terms of postoperative morbidity and recovery and long-term functional outcomes and quality of life [8]. While TEM has revolutionised technique and outcome of transanal surgery, becoming the ‘gold standard’ for the treatment of large rectal adenomas [9–12], concerns remain about its role in the treatment of rectal cancer, mainly due to the lack of adequate lymphadenectomy. This manuscript aims to review current indications and future perspectives of TEM.

## SURGICAL TECHNIQUE

In many centers, a TEM procedure is now performed with TEO® (transanal endoscopic operation) instrumentation by Karl Storz GmbH (Tuttlingen, Germany). The equipment includes a 7 or 15 cm rectal tube which has a 4 cm diameter and three working channels (12, 5 and 5 mm) for dedicated or conventional laparoscopic instruments and a 5 mm channel for a 30° 2D scope. The proctoscope is connected to the operating table via a holding arm consisting of three joints and a single screw. The system is used in combination with standard laparoscopic units. Camera imaging is projected on screen and insufflation is obtained by a conventional CO_2_ thermo-insufflator. The shape of the tip of the proctoscope allows manipulation and suturing of the rectal wall on a 360° surface.

Recently, transanal minimally invasive surgery (TAMIS), using equipment for single-incision laparoscopic surgery (SILS), has been proposed as an alternative to TEM. Indications and surgical technique are the same as for TEM [13]. There is a lower cost for the disposable SILS equipment, compared with the reusable TEM device. However, no comparative studies aimed at evaluating the benefits in terms of costs and clinical outcome of TAMIS versus TEM in high volume centers over long periods of time have been published.

The TEM procedure is usually performed under general anesthesia. The patient is placed either prone or supine in order to keep the lesion as close to the 6 o’clock position as possible. Patients with lateral lesions are usually placed in the supine position unless the lesion is predominantly located in the right or left upper quadrant (i.e. 12 to 3-, or 9 to 12 o’clock position). With circumferential lesions, the patient is always positioned prone due to the higher risk of entering the peritoneal cavity and the consequent need to reduce the descent of small bowel loops into the surgical field while repairing the opening itself.

After insertion of the proctoscope, the lesion is identified and the proctoscope is fixed. Its position is adjusted throughout the procedure in order to ensure optimal visualization and access to the margins of the lesion. High-flow CO_2_ insufflation is required and endoluminal pressure is generally maintained at 8 mmHg, although it might need to be increased to 16 mmHg.

Dissection usually begins at the right lower border of the tumor. A macroscopic margin of at least 5 mm from the neoplasm needs to be obtained with both benign and malignant lesions. Tumor excision is performed by monopolar hook cautery. In difficult cases, ultrasonic shears or an electrothermal bipolar vessel sealing system may be helpful. Dissection is continued circumferentially around the lesion to the perirectal fat. Due to the uncertainty of the pre-operative diagnosis and staging, full-thickness resection with adequate margins of clearance should be performed. The specimen is retrieved transanally and is pinned on a corkboard before fixation in 10% buffered formalin, in order to preserve the margins of normal mucosa surrounding the tumor. The specimen is analysed by permanent section.

After disinfection of the parietal defect with iodopovidone solution, the rectal wall is always closed with one or more Maxon 3-0 (Codisan® S.p.A.) running sutures secured with dedicated silver clips (Richard Wolf, Knittingen, Germany). These clips serve to anchor the suture in place, since knot tying during TEM is challenging. As an alternative, the Endo Stitch™ single-use suturing device can also be used. At this stage, the endoluminal pressure may be reduced to allow better compliance of the rectal wall. Suturing is performed with particular attention to the integrity of the rectal lumen. Therefore, when suturing large defects, a midline stitch is placed to approximate proximal and distal margins. At the end of the procedure, patency of the rectum is carefully verified through the TEM proctoscope.

## CURRENT INDICATIONS

### TEM for rectal adenomas

Endoscopic resection represents the treatment of choice for pre-malignant lesions of the gastrointestinal tract. However, conventional endoscopic mucosal resection (EMR) cannot provide an *en-bloc* resection in case of large lesions and incomplete or piecemeal resection may occur in up to 50% of cases [14]. After piecemeal resection, pathological assessment of complete resection is challenging and the risk of local recurrence is high [15]. In addition, EMR does not provide a submucosal dissection, therefore precluding an accurate staging in case of malignancy.

In the last few years, the endoscopic submucosal dissection (ESD) technique was introduced to overcome these difficulties and to allow *en bloc* resection of specimens, especially in case of lesions larger than 20 mm [16]. Low complication rates and low local recurrence rates have been reported after ESD [17–19]; however, compared with conventional EMR, ESD is technically more challenging and time consuming, requiring a steep learning curve [17, 20]. As a result, ESD has not gained wide acceptance in western countries and transanal surgery is still the approach of choice for the excision of large rectal adenomas.

Today, no studies have compared endoscopic techniques with transanal surgery for large rectal adenomas. Barendse *et al.* [21] published a systematic review on safety and effectiveness of EMR versus TEM for large rectal adenomas, including 20 prospective and non-prospective case series employing EMR technique and 48 employing TEM technique with similar follow-up periods. Postoperative complication rates were 3.8% for EMR vs 13.0% for TEM (*P* < 0.001). Local recurrence rates were assessed in 3890 patients (1030 EMR and 2860 TEM). Early local recurrence after single intervention in the EMR series was significantly higher than in the TEM series (11.2 vs 5.4%, respectively; *P = *0.04), while late recurrence rates were similar in both groups of patients: 1.5% for EMR vs 3.0% for TEM (*P = *0.29). The authors concluded that EMR for large rectal adenomas appears to be less effective but safer than TEM.

However, because of the low quality of the studies included in this analysis, no definitive conclusions can be drawn and the results of prospective randomized trials are needed to assess the role of TEM compared with EMR/ESD in the treatment of rectal adenomas.

Several transanal techniques for excision of rectal polyps unsuitable for endoscopic resection have been described, including conventional transanal resection (TE) with retractors and TEM [22]. Local recurrence rates range from 4–57% after TE and from 2.4–16% after TEM [23].

Several studies have compared conventional TE to TEM for adenoma, reporting significantly better long-term results after TEM. For instance, Langer *et al.* retrospectively compared the long-term outcomes of 54 patients undergoing conventional TE and 57 patients undergoing a TEM procedure for rectal adenoma [24]. They reported a significantly higher local recurrence rate after TE than after TEM (31.5 vs 8.8%, respectively). Similar results were published by Moore *et al.*, who reviewed the outcomes of 40 patients undergoing TEM and 38 undergoing TE [25]. They reported a significantly higher rate of negative margins (83 vs 61%; *P = *0.03), a trend toward a reduced rate of specimen fragmentation (12% vs 26%; *P = *0.12) and a significantly lower local recurrence rate (3 vs 32%; *P = *0.003) after TEM, compared with TE. Finally, the findings reported by de Graaf *et al.* support the superiority of TEM over classical TE with regard to surgical margins status, specimen fragmentation and local recurrence [26]. They observed negative resection margins in 88% of specimens after TEM compared with 50% after TE (*P < *0.001), fragmentation of the specimen in 1.4% of case after TEM and 23.8% after TE (*P < *0.001) and local recurrence rate of 6.1% after TEM, compared with 28.7% after TE (*P < *0.001).

Therefore, based on the data reported in the literature, TEM represents the current standard of treatment for large rectal adenomas and conventional TE should be abandoned.

Residual adenomatous tissue is detected in the surgical margins in 0–37% of TEM procedures and positive surgical margins are independent risk factors for local recurrence [12, 27]. Despite such high positive-residual-margin rates, reported recurrence rates are significantly lower, ranging from 3–16% [23]. This could be explained by the fact that diathermic damage to the remaining adenomatous tissue during the dissection may cause the sterilization of the margins.

Another risk factor for local recurrence is the size of the adenoma. In our clinical practice, a full-thickness incision of the rectal wall is always initiated at a distance of approximately 5 mm around the tumor. Nevertheless, in our series of 293 large rectal adenomas treated by TEM [12], 21% of adenomas with a diameter ≥5 cm were removed with positive margins, versus 9% of adenomas <5 cm (*P = *0.047). Tumor diameter ≥5 cm was found to be a predictive factor for local recurrence (*P = *0.007).

Our results compare favorably with those reported by McCloud *et al.* in a series of 75 patients undergoing TEM for adenoma [28]. They found a significantly higher local recurrence rate at 12 months for adenomas larger than 10 cm, compared with those with a diameter between 5 and 10 cm and those smaller than 5 cm (33.3 vs 21.7 vs 7.7%, respectively; *P = *0.035). Similarly, Scala *et al.* recently looked at the outcomes of 279 TEM procedures performed for benign lesions [29], reporting significantly increased local recurrence rates for lesions larger than 5 cm.

Since a local recurrence is relatively common after excision of adenomas larger than 5 cm, a strict clinical and endoscopic follow-up is highly recommended in these cases. However, TEM has been shown to be an important therapeutic option even in the treatment of recurrent adenoma, when the endoscopic resection is not feasible. Several series have reported on the safety and effectiveness of TEM in the treatment of recurrent adenoma and no increased perioperative morbidity and no further cases of local recurrence have been described [11, 12, 28, 30–33].

Finally, in 2012 there are still some limitations in the pre-operative diagnosis of large rectal adenomas. Even though EUS appears to be the most accurate pre-operative diagnostic tool for investigating tumor invasion of the rectal wall, discrepancy rates up to 20% between pre-operative EUS and pathological staging of the tumors are reported. Some recent studies have investigated the role of EUS, compared with magnetic resonance imaging (MRI), for the staging of large rectal adenomas, reporting similar rates of over-staging (21.7%) between the two staging tools. However, MRI might be more appropriate in case of more proximal rectal tumors that cannot be easily reached by the EUS probe [34]. In addition, up to 26% of the adenomas resected by TEM are found, at the definitive pathological examination, to be invasive adenocarcinoma [35]. With this in mind, an appropriate full-thickness excision should be offered to all patients with rectal neoplasm, even in case of benign pre-operative histology, instead of a partial wall, piecemeal endoscopic resection. This strengthens the concept of TEM as a macrobiopsy that is radical in case of low-risk pT1 cancers, while it represents the first step in a multidisciplinary strategy for the treatment of more advanced rectal cancer that includes rectal resection and TME and chemoradiation therapy.

### TEM for T1 rectal cancer

While TEM is considered the primary form of treatment for large rectal adenomas judged unsuitable for endoscopic removal, its role in the treatment of early rectal cancer (T1) is still controversial, mainly because of the absence of an adequate lymphadenectomy.

To date, rectal resection with TME is the ‘gold standard’ in the treatment of extraperitoneal rectal cancer [36, 37]. However, local recurrence can develop even after a radical resection with complete TME for T1 N0 rectal cancer[38]. In addition, abdominal surgery is associated with significant mortality and morbidity, including anastomotic leaks, urinary and sexual dysfunction and fecal incontinence [4, 7].

With the widespread introduction of population-based screening programs, the incidence of early rectal cancer has progressively risen during the last twenty years, leading to an increasing scrutiny and debate around the potential role of TEM in the treatment of early rectal cancer.

A recent meta-analysis of the literature analysed the short-term and oncological outcomes of the single randomized clinical trial and four retrospective, comparative, non-randomized studies published between 1996 and 2009, that have compared TEM to rectal resection with TME for T1 rectal cancer [39]. Globally, a significantly lower postoperative complication rate was reported after TEM, compared with TME (8.2 vs 47.2%; *P = *0.01), with no mortality, confirming the safety of TEM, even in the treatment of early rectal cancers.

A significantly higher local recurrence rate was found after TEM (12 vs 0.5%; *P = *0.004). However, the wide range of local recurrence rates from 4–18% observed after TEM for T1 rectal cancer in these studies can be explained by several factors: i) heterogeneity of the studies, which were often underpowered and had extremely variable follow-up periods, ii) different inclusion criteria and iii) lack of differentiation between ‘low risk’ (well or moderately differentiated adenocarcinoma without lymphatic invasion) or ‘high-risk’ carcinoma (poorly or undifferentiated adenocarcinoma with lymphatic invasion) in the majority of them.

The only two comparative studies that have analysed long-term outcomes of TEM for T1 rectal cancers, classified according to Hermanek criteria, are those published by Heintz *et al.* in 1998 and by Lee *et al.* in 2003 [40, 41]. Heintz *et al.* did not observe significant differences in terms of local recurrence after TEM compared with TME (4 vs 3%) in case of a T1 low-risk cancer, while a local recurrence was more frequent after TEM than TME in case of a ‘high risk’ cancer (33 vs 18%) [40]. Similar results were obtained by Lee *et al.* in 52 patients who had undergone TME, compared with 17 patients treated by TME for well- or moderately differentiated rectal carcinomas [41]. They found comparable local recurrence rates (4 vs 0%; *P = *0.95).

Since the early 1990s, several case series have been published, assessing oncological outcomes after TEM for T1 rectal cancer [42]. Reported recurrence rates ranged from 0–26%. During the last decade, several risk factors for local recurrence after TEM for T1 rectal cancer have been evaluated, other than the degree of tumor differentiation and the lympho-vascular invasion. They include positive resection margins, the tumor diameter and the T-stage according to submucosal invasion [43, 44].

A tumor diameter >4 cm is usually considered a risk factor for recurrence after local excision, as it is associated with an increased rate of positive margins. However, tumor involvement of the resection margins in T1 cancers is occasional (about 2%) even in cases of large tumors, when a full-thickness excision is performed [44]. TEM with a full-thickness excision allows reduction of the rate of positive deep margins, while the circumferential mucosal margins are easily marked before beginning the excision.

Submucosal (sm) invasion is one of the strongest predictors of lymph node metastasis and local recurrence along with the lympho-vascular invasion. The incidence of lymph node metastasis is very low for T1 sm1 (0 to 3%), but increases to 15 and 25% for T1 sm2–3 and T2, respectively. Several studies have specifically looked at the significance of submucosal invasion as prognostic factor for recurrence after TEM for T1 rectal cancer [43, 44]. For instance, Bach *et al.* [43] used the oncological outcomes, prospectively collected in a multicenter database of 487 rectal cancer patients (253 pT1) treated by TEM, to construct a predictive model of local recurrence after TEM. They found that depth of submucosal invasion >sm1 was an independent predictor of local recurrence, while the risks of recurrence for sm2–3 and pT2 lesions were similar.

We recently reviewed our series of 107 patients undergoing TEM for rectal cancer. Among the 48 pT1 cancer patients, during a mean follow-up of 54 months, the overall recurrence rate was 10.4%. None of the 26 patients with an sm1 lesion experienced a local recurrence, while a local recurrence occurred in 5 out of 22 (22.7%) sm2–3 patients (*P = *0.036). Sm1 lesions showed a 100% disease-free rate at 60 months. By multivariate analysis, sm staging was an independent predictor for recurrence, along with the tumor grading [44].

Based on the evidence reported in the literature, it seems evident that an accurate pre-operative evaluation of the depth of tumor invasion and lymph node metastasis is crucial for proper patient selection for a TEM procedure. EUS appears to be the most accurate pre-operative diagnostic tool for investigating the tumor invasion of the rectal wall [45]. However, EUS is highly operator-dependent and several factors, including previous endoscopic biopsies, endoscopic manipulation of the tumor and peritumoral inflammation, may affect the accuracy of the evaluation of T1 rectal lesions [46]. Therefore, a discrepancy between pre-operative EUS and definitive pathological staging of the tumor is quite common, with a risk of under-staging that is as high as 25% and a risk of over-staging up to 20%. Nevertheless, the recent introduction of high-definition 20 Mhz through-the-channel mini-probes may permit better pre-operative identification of not only the T-staging, but also the depth of submucosal invasion [47].

High-resolution MRI is less operator-dependent and is widely used for the pre-operative staging of rectal cancer. While EUS better shows an early rectal cancer, differentiating accurately between T1 and T2 rectal cancer, MRI is more accurate in the detection of mesorectal invasion and in the evaluation of the distance to the mesorectal fascia [48, 49]. With this in mind, TEM with full-thickness excision should be used as macrobiopsy and considered a means for staging early rectal cancer.

Finally, when the definitive pathological evaluation of the TEM specimen reveals the presence of negative prognostic factors—such as depth of tumor invasion beyond pT1 sm1, poorly differentiated tumor grading, lympho-vascular invasion or positive resection margins—abdominal surgery with TME is recommended in order to reduce the risk of recurrence. There is increasing evidence suggesting that TEM used as macrobiopsy does not jeopardize long-term survival of patients who undergo further abdominal surgery. For instance, Borschitz *et al.* have retrospectively compared the oncological outcome of 17 pT1 ‘high risk’ patients undergoing abdominal rectal surgery after TEM to 66 pT1 ‘low risk’ patients who were treated by TEM alone [50]. They reported a local recurrence rate of 6% in both groups. Five-year cancer-free survival was 93% in the ‘high risk’ patients, compared with 94% in ‘low risk’ patients. Recently, Levic *et al.* have reported similar local recurrence and distant metastasis rates in a case-matched study that compared patients undergoing a TME after a TEM procedure to patients treated with primary TME for rectal cancer (0 vs 8% and 4 vs 12%, respectively [51].

## FUTURE PERSPECTIVES

### Neoadjuvant combined-modality therapy and TEM for T2 N0 rectal cancer

Modern neoadjuvant chemoradiation therapy has been shown to induce a significant tumor regression, down-staging and sterilization of perirectal lymph nodes, with a pathological complete response that is reported in up to 30% of patients [52]. A recent systematic review and meta-analysis of the literature has demonstrated that oncological outcomes following pathological complete response are significantly better, compared with non-responders, in terms of local recurrence rate, distant metastasis rate and both overall and disease-free survival at 5 years [53]. Among the many factors that are implicated in tumor regression, the interval between completion of neoadjuvant treatment and surgery seems to play a key role. In particular, pathological complete response appears to be a time-dependent process [53].

TME is associated with mortality and significant postoperative short- and long-term morbidity, including sexual and urinary dysfunction and stoma-related complications [4–7, 54, 55]. Since 30% of patients may have a pathological complete response and they will be subjected to an ‘unnecessary’ procedure associated with significant short-term and long-term morbidity, there is an increasing interest in properly identifying these patients in order to be able to offer a less invasive—and still oncologically adequate—treatment. In 2004, Habr-Gama *et al.* [56] published the long-term results of a retrospective study in which 71 patients (14 T2 and 49 T3) with clinical complete response after long-course chemoradiation therapy (observation group) were compared with 22 patients who had undergone surgery for incomplete clinical response and had a final diagnosis of pT1 N0 M0 (resection group). Among the observation group, after a mean follow-up of 57.3 months, luminal recurrence occurred in 2.8% of patients, while distant metastasis developed in 4.2%. No pelvic recurrences were reported. Five-year overall and disease-free survival rates were 100% and 92%, respectively. In the resection group, three patients developed distant metastasis, while no luminal or pelvic recurrences occurred. Five-year overall and disease-free survival rates were 88% and 83%, respectively. Based on these results, a ‘watch and wait’ strategy was proposed in patients with clinical complete response. However, in a follow-up study from the same group, that included 122 patients who were initially considered to have a complete clinical response and therefore managed conservatively [57], the authors reported a local recurrence in 23 patients (18.9%), during the first 12 months. After a mean follow-up of 59.9 months, among the 99 patients included, luminal recurrence rate was 5% and distant metastasis occurred in 7% of patients. Overall and disease-free 5-year survivals were 93% and 85% respectively.

These data highlight the challenge of identifying patients with a durable, complete clinical response. A correlation, between tumor invasion of the rectal wall after neoadjuvant chemoradiation and the risk of lymph node metastasis has been observed, ranging from 2–17% in ypT0-1 to 48% in ypT3-4 patients [58, 59]. More recent studies with longer intervals between completion of neoadjuvant treatment and surgery have reported an incidence of lymph node metastasis in ypT0 under 5% [60–63].

Reliable assessment of the rectal wall and nodal status after chemoradiation remains challenging. Accuracy of the available staging modalities is disappointing, due to the radiation-induced fibrosis, edema and inflammation. In addition, radiotherapy has been known to reduce both the number and size of the perirectal lymph nodes [64].

In addition, the clinical correlation between complete clinical response and pathological response is poor. Smith *et al.* evaluated the clinical significance of residual mucosal abnormalities after neoadjuvant therapy for rectal cancer in 220 patients [65]. The diameter of residual mucosal abnormalities correlated statistically with pathological tumor stage, which was in turn associated with pathological nodal status and lymph node ratio. Lymph node metastasis were retrieved in only one patient (4.2%) staged as ypT0-1 and the risk of nodal metastasis was associated with poor tumor differentiation and lympho-vascular invasion. Interestingly, more than 50% of patients with a complete pathological response did not have a complete clinical response: residual mucosal abnormalities less than 3 cm were strongly associated with ypT0-1 and a low rate (2%) of lymph node metastasis. The authors concluded that this subgroup of patients could be offered local excision as a macrobiopsy, to rule out the persistence of cancer within the rectal wall and to avoid the risks of an unnecessary TME.

The role of local excision after neoadjuvant chemoradiation has been evaluated by several retrospective series and reviewed in a pooled analysis by Borschitz *et al.* who included 273 patients from seven different series [66]. They showed that the risk of local recurrence was strictly correlated with the pathological staging observed after chemoradiation. The strongest prognostic factors were ypT0 (0% of local recurrence) and ypT1 (2%), while ypT2 was associated with increasing local recurrence rates of 6–20%, with a mean rate of 7%. This wide range of local recurrences among ypT2 patients has to be interpreted with caution, as ‘low risk’ ypT2 (G1-2, without lympho-vascular invasion) may have a different clinical behavior, compared with ‘high risk’ ypT2 (G3 and/or lympho-vascular invasion). Not surprisingly, patients with no pathological response (ypT3) showed a risk of local recurrence up to 42%.

Nowadays, TEM is proposed as an integral component of the multi-modality treatment of high selected T2 N0 rectal cancers. TEM equipment allows for stable exposure of the surgical field, adequate assessment of the margin and minimal risk of piecemeal excision, tumor fragmentation and seeding.

Lezoche *et al.* randomly assigned to TEM—or to rectal resection combined with TME—70 patients with a pre-operatively staged T2N0M0 G1-2 rectal cancer with a diameter less than 3 cm after neoadjuvant combined modality therapy [67]. A 30% rate of complete pathological response was reported (32% in the TEM group and 29% in the laparoscopic group). No differences were observed in terms of local recurrences and survival between the two groups at a median follow-up of 84 months. Notably, all recurrences occurred in patients without significant response to neoadjuvant chemoradiation.

Recently, Garcia-Aguilar *et al.* reported the preliminary results of the American College of Surgeons Oncology Group (ACOSOG) Z6041 trial [68], looking at short-term outcomes of neoadjuvant chemoradiation followed by local excision—performed by conventional transanal technique or TEM—for treatment of 77 patients with a clinically staged T2 N0 rectal cancer [68]. A complete pathological response was achieved in 34 patients (44%), while down-staging was observed in 49 patients (64%).

We recently reviewed the oncological outcomes of 43 patients who had undergone TEM for a T2 N0 rectal cancer. Among these patients, 11 underwent pre-operative radiotherapy. A response to radiation therapy in terms of downsizing was observed in nine patients (82%), who were then were treated by TEM while, in 2 two cases (18%), a local tumor progression was observed. During a median follow-up of 70 months (range: 36–140), all nine patients with a downsized rectal cancer were alive and disease-free, while both patients who had progression of the disease died of distant metastasis [69].

While evidence supporting the role of TEM in a multidisciplinary strategy for the treatment of rectal cancer is slowly increasing, concerns have recently been raised regarding the healing process in patients undergoing TEM after neoadjuvant chemoradiation. Complication rates related to the rectal wound range from 0–60.9% [69, 70]. Marks *et al.* compared short-term outcomes of 43 rectal cancer patients, treated by neoadjuvant chemoradiation therapy followed by TEM (XRT group), with those of 19 patients treated by TEM alone [71]. The overall morbidity rate was significantly higher in the XRT group (33 vs 5.3%; *P* < 0.05). In particular, the wound complication rate was 25.6% for the XRT group (11 cases) and 0% for the non-XRT group (*P = *0.015). However, ten patients (91%) were treated conservatively and only one patient required a diverting colostomy.

Perez *et al.* reported the 30-day results of 36 consecutive patients treated by TEM for rectal neoplasm [70]: 23 underwent chemoradiation therapy followed by TEM and 13 were managed by TEM alone for adenomas (four cases), early adenocarcinomas (six patients), carcinoids (two cases) and gastrointestinal stromal tumor (one patient). They reported a significantly higher rate of suture line dehiscence (60.9 vs 23.1%; *P = *0.032) and hospital readmission (43.5 vs 7%; *P = *0.025) among patients who had undergone TEM after neoadjuvant chemoradiation. However, no patient in this series required operative treatment to repair the dehiscence. In addition, no differences were observed in terms of late complications: one patient in each group developed a symptomatic rectal stenosis that required rectal dilation.

Based on the data currently available, even though neoadjuvant treatment seems to increase the rate of wound-related complications after TEM, larger studies with long follow-up periods are needed to evaluate the risk of late complications and understand the implications in terms of oncological outcomes. A European multicenter, prospective study, Transanal Endoscopic Microsurgery After Radiochemotherapy for Rectal Cancer (CARTS) has been designed to investigate the role of TEM performed 8–10 weeks after pre-operative treatment on the basis of clinical response [72]. The short-term and the oncological results of this study, along with those of ACOSOG Z6041, may allow us to draw more definitive conclusions regarding the role of transanal excision in the treatment of locally advanced rectal cancer. Therefore, we feel that, at present, TEM should be proposed for the treatment of T2 N0 rectal cancer only in the context of study protocols after approval by the local ethical committee.

Overall, there is increasing evidence that TEM may play a major role in the multidisciplinary management of highly selected T2 N0 rectal cancer patients with a significant response to neoadjuvant therapy or a complete pathological response. Transanal excision—and full-thickness TEM specifically—should be considered a ‘staging’ biopsy, to allow for a pathological evaluation of the specimen. Further decisions regarding the surgical management (‘watch and wait’ vs TME) should be made on the basis of the pathological evaluation, imaging study and tumor characteristics.

### TEM and NOTES

In the NOTES (Natural Orifice Transluminal Endoscopic Surgery) era, transrectal access to the peritoneal cavity has been variously described [73–75]. Main concerns are the safety of the access and the closure of the transvisceral enterotomy. TEM has been proposed as a platform for NOTES, since it is a well-established technique that allows both full-thickness resection and suture of the rectal wall defect. The feasibility of some transrectal NOTES procedures (diagnostic peritoneoscopy, liver biopsy, sigmoid resection) using TEM instrumentation, suggesting TEM as a portal for NOTES, has been recently demonstrated in experimental studies [76].

Even though satisfactory results have been achieved in experimental (animal and human cadaver) models, the potential clinical consequences of a transrectal NOTES procedure in humans, in terms of intra-abdominal contamination, leak of the enterotomy and risk of a stoma, are poorly evaluated.

To our knowledge, only one case of NOTES transanal rectal cancer using TEM and laparoscopic assistance has been published [77]. Transanal endoscopic rectal resection with TME using the TEM platform was performed in a 76-year-old woman after neoadjuvant chemoradiotherapy for a T2N2 mid-rectal cancer. No postoperative complications occurred. The final pathological evaluation demonstrated a complete TME with negative resections margins. However, no long-term results in terms of recurrence and survival are available.

In order to better clarify the effects of a peritoneal perforation (PP) during TEM on short-term and oncological outcomes, we have recently reviewed our series of PP during TEM for rectal neoplasm and compared the clinical outcomes with the data available in the literature [78]. In our experience of 481 patients, PP occurred in 28 cases (5.8%). The indications for TEM were 23 adenomas and 5 carcinomas. PP was sutured by TEM in 25 cases (89.3%), while conversion to abdominal surgery was necessary in 3 cases (10.7%). Notably, all conversions occurred during the first 100 TEM procedures. By multivariate analysis, the tumor distance ≥7 cm from the anal verge (*P = *0.010) was the only independent predictor for PP. The operative time was significantly longer in case of intra-operative PP than in uneventful TEM, while postoperative morbidity rate (3.6 vs 6.2%) and type of complications were similar in both groups. No mortality occurred in the series.

Globally, 17 studies have reported the number of PP occurrences during TEM, with a mean PP rate of 4.8% and a range from 0–32.3%, reflecting the fact that a submucosal dissection may be preferred over a full-thickness excision in cases at risk for PP by some surgeons [78]. The learning curve and the case volume of the surgeon are two main factors that influence the treatment strategy to be adopted when PP occurs. Notably, conversion to open surgery was reported in 50–100% cases of PP only in series with less than 100 patients, whereas it ranged from 0–40% in larger series.

These data confirm the results obtained by Salm *et al.* in a survey of 1900 TEM procedures [79]. They reported that the conversion rate to laparotomy during TEM for all causes, including inadvertent transrectal opening of the peritoneal cavity, decreased with experience from 11.6% (1–10 TEM procedures) to 1.2% (>100 TEM procedures).

In the short-term period, no cases of pelvic sepsis or infectious complications after PP have been reported, suggesting the fact that TEM seems not to be associated with a higher risk of pelvic infection or other complications when a PP occurs. Furthermore, the low morbidity rate and the absence of pelvic infection complications demonstrate that a defunctioning stoma is not generally necessary in high-volume institutions.

Insufflation of CO_2_ from the rectum into the peritoneum is considered a potential cause of oncological complications in patients with colorectal cancer. At present, very few data are available about oncological outcomes after PP during TEM. To our knowledge, the only study to evaluate the oncological results of patients undergoing TEM with an inadvertent PP was that by Baatrup *et al.* [80], who reported 22 perforations into the peritoneal cavity during a total of 888 TEM procedures for rectal cancer, performed at four European centers. During a median follow-up of 36 months (range: 3–164), local recurrence developed in one pT1 patient (7%) and in one pT2 patient (25%), while distant metastasis were detected in three patients.

In our series, over a median follow-up period longer than 4 years, all patients in whom a PP occurred during TEM for adenoma or pT1 rectal cancer are disease-free, with no sign of intraperitoneal seeding of adenomatous or cancer tissue.

## CONCLUSIONS

In 2013, TEM is the safest and most effective treatment modality available for large rectal adenomas, with significantly higher complete resection rates and lower local recurrence rates than conventional transanal excision. Further studies are needed to evaluated the safety and efficacy of TEM compared with EMR/ESD.

Non-ulcerated rectal cancers, with the tumor invasion confined to the superficial submucosa (i.e. pT1 sm1), well or moderately differentiated, without lymphovascular invasion, are the only malignant lesions currently suitable for TEM. In this highly select group of cancers, TEM alone provides oncological outcomes that approximate to those of abdominal surgery.

For pT1 sm2–3 and pT2 cancers, TEM as sole treatment modality is not recommended because of the high risk of lymph node metastasis. Future studies are needed to investigate the role of TEM in association with neoadjuvant therapy in this subgroup of patients. Furthermore, we are at present investigating the technical possibility of performing sentinel lymph node sampling in oncological TEM procedures.

In the NOTES era, we feel that the application of the transanal approach to NOTES should be limited only to selected academic centers with extensive expertise in TEM. Large studies with long follow-up periods are requested before this approach can be widely applied to the treatment of colorectal cancer.

**Conflict of interest:** none declared.

## References

[1] Buess G, Hutterer F, Theiss R (1984). Das System für die transanale endoskopische Rektumoperation. Chirurg.

[2] Sakamoto GD, MacKeigan JM, Senagore AJ (1991). Transanal excision of large, rectal villous adenomas. Dis Colon Rectum.

[3] Mellgren A, Sirivongs P, Rothenberger DA (2000). Is local excision adequate therapy for early rectal cancer?. Dis Colon Rectum.

[4] Morino M, Parini U, Giraudo G (2003). Laparoscopic total mesorectal excision: a consecutive series of 100 patients. Ann Surg.

[5] Morino M, Parini U, Allaix ME (2009). Male sexual and urinary function after laparoscopic total mesorectal excision. Surg Endosc.

[6] Wallner C, Lange MM, Bonsing BA, Cooperative Clinical Investigators of the Dutch Total Mesorectal Excision Trial (2008). Causes of fecal and urinary incontinence after total mesorectal excision for rectal cancer based on cadaveric surgery: a study from the Cooperative Clinical Investigators of the Dutch total mesorectal excision trial. J Clin Oncol.

[7] Hendren SK, O'Connor BI, Liu M (2005). Prevalence of male and female sexual dysfunction is high following surgery for rectal cancer. Ann Surg.

[8] Allaix ME, Rebecchi F, Giaccone C (2011). Long-term functional results and quality of life after transanal endoscopic microsurgery. Br J Surg.

[9] Ramirez JM, Aguilella V, Gracia JA (2009). Local full-thickness excision as first line treatment for sessile rectal adenomas: long-term results. Ann Surg.

[10] de Graaf EJ, Doornebosch PG, Tetteroo GW (2009). Transanal endoscopic microsurgery is feasible for adenomas throughout the entire rectum: a prospective study. Dis Colon Rectum.

[11] Guerrieri M, Baldarelli M, de Sanctis A (2010). Treatment of rectal adenomas by transanal endoscopic microsurgery: 15 years' experience. Surg Endosc.

[12] Allaix ME, Arezzo A, Cassoni P (2012). Recurrence after transanal endoscopic microsurgery for large rectal adenomas. Surg Endosc.

[13] Albert MR, Atallah SB, Debeche-Adams TC (2013). Transanal minimally invasive surgery (TAMIS) for local excision of benign neoplasms and early-stage rectal cancer: efficacy and outcomes in the first 50 patients. Dis Colon Rectum..

[14] Puli SR, Kakugawa Y, Gotoda T (2009). Meta-analysis and systematic review of colorectal endoscopic mucosal resection. World J Gastroenterol.

[15] Iishi H, Tatsuta M, Iseki K (2000). Endoscopic piecemeal resection with submucosal saline injection of large sessile colorectal polyps. Gastrointest Endosc.

[16] Yamamoto H, Kawata H, Sunada K (2003). Successful en-bloc-resection of large superficial tumors in the stomach and colon using sodium hyaluronate and small-caliber-tip-transparent hood. Endoscopy.

[17] Probst A, Golger D, Arnholdt H (2009). Endoscopic submucosal dissection of early cancers, flat adenomas and submucosal tumors in the gastrointestinal tract. Clin Gastroenterol Hepatol.

[18] Fujishiro M, Yahagi N, Nakamura M (2006). Endoscopic submucosal dissection for rectal epithelial neoplasia. Endoscopy.

[19] Onozato Y, Kakizaki S, Ishihara H (2007). Endoscopic submucosal dissection for rectal tumors. Endoscopy.

[20] Kakushima N, Fujishiro M, Kodashima S (2006). A learning curve for endoscopic submucosal dissection of gastric epithelial neoplasms. Endoscopy.

[21] Barendse RM, van den Broek FJC, Dekker E (2011). Systematic review of endoscopic mucosal resection versus transanal endoscopic microsurgery for large rectal adenomas. Endoscopy.

[22] Parks AG (1968). A technique for excising extensive villous papillomatous change in the lower rectum. Proc R Soc Med.

[23] Casadesus D (2009). Surgical resection of rectal adenoma: a rapid review. World J Gastroenterol.

[24] Langer C, Liersch T, Süss M (2003). Surgical cure for early rectal carcinoma and large adenoma: transanal endoscopic microsurgery (using ultrasound or electrosurgery) compared with conventional local and radical resection. Int J Colorectal Dis.

[25] Moore JS, Cataldo PA, Osler T (2008). Transanal endoscopic microsurgery is more effective than traditional transanal excision for resection of rectal masses. Dis Colon Rectum.

[26] de Graaf EJR, Burger JWA, van Ijsseldijk ALA (2011). Transanal endoscopic microsurgery is superior to transanal excision of rectal adenomas. Colorectal Dis.

[27] Whitehouse PA, Tilney HS, Armitage JN (2006). Transanal endoscopic microsurgery: risk factors for local recurrence of benign rectal adenomas. Colorectal Dis.

[28] McCloud JM, Waymont N, Pahwa N (2006). Factors predicting early recurrence after transanal endoscopic microsurgery excision for rectal adenoma. Colorectal Dis.

[29] Scala A, Gravante G, Dastur N (2012). Transanal endoscopic microsurgery in small, large and giant rectal adenomas. Arch Surg.

[30] Katti G (2004). An evaluation of transanal endoscopic microsurgery for rectal adenoma and carcinoma. JSLS.

[31] Vorobiev GI, Tsarkov PV, Sorokin EV (2006). Gasless transanal endoscopic surgery for rectal adenomas and early carcinomas. Tech Coloproctol.

[32] Guerrieri M, Baldarelli M, Morino M (2006). Transanal endoscopic microsurgery in rectal adenomas: experience of six Italian centers. Dig Liver Dis.

[33] Platell C, Denholm E, Makin G (2004). Efficacy of transanal endoscopic microsurgery in the management of rectal polyps. J Gastroenterol Hepatol.

[34] De Vargas Macciucca M, Casale A, Manganaro L (2010). Rectal villous tumors: MR features and correlation with TRUS in the pre-operative evaluation. Eur J Radiol.

[35] Léonard D, Colin JF, Remue C (2012). Transanal endoscopic microsurgery: long-term experience, indication expansion and technical improvements. Surg Endosc.

[36] MacFarlane JK, Ryall RDH, Heald RJ (1993). Mesorectal excision for rectal cancer. Lancet.

[37] Heald RJ, Moran BJ, Ryall RDH (1998). The Basingstoke experience of total mesorectal excision, 1978–1997. Arch Surg.

[38] Peeters KC, Marijnen CA, Nagtegaal ID, Dutch Colorectal Cancer Group (2007). The TME trial after a median follow-up of 6 years: increased local control but no survival benefit in irradiated patients with resectable rectal carcinoma. Ann Surg.

[39] Wu Y, Wu YY, Zhu BS (2011). TEM and conventional rectal surgery for T1 rectal cancer: a meta-analysis. Hepatogastroenterology.

[40] Heintz A, Mörschel M, Junginger T (1998). Comparison of results after transanal endoscopic microsurgery and radical resection for T1 carcinoma of the rectum. Surg Endosc.

[41] Lee W, Lee D, Choi S (2003). Transanal endoscopic microsurgery and radical surgery for T1 and T2 rectal cancer. Surg Endosc.

[42] Suppiah A, Maslekar S, Alabi A (2008). Transanal endoscopic microsurgery in early rectal cancer: time for a trial?. Colorectal Dis.

[43] Bach SP, Hill J, Monson JR, Association of Coloproctology of Great Britain and Ireland Transanal Endoscopic Microsurgery (TEM) Collaboration (2009). A predictive model for local recurrence after transanal endoscopic microsurgery for rectal cancer. Br J Surg.

[44] Morino M, Allaix ME, Caldart M (2011). Risk factors for recurrence after transanal endoscopic microsurgery for rectal malignant neoplasm. Surg Endosc.

[45] Puli SR, Bechtold ML, Reddy JB (2009). How good is endoscopic ultrasound in differentiating various T-stages of rectal cancer? Meta-analysis and systematic review. Ann Surg Oncol.

[46] Doornebosch PG, Bronkhorst PJ, Hop WC (2008). The role of endorectal ultrasound in therapeutic decision-making for local vs transabdominal resection of rectal tumors. Dis Colon Rectum.

[47] Zhou PH, Yao LQ, Zhong YS (2004). Role of endoscopic miniprobe ultrasonography in diagnosis of submucosal tumor of large intestine. World J Gastroenterol.

[48] Beets GL, Beets-Tan RG (2010). Pretherapy imaging of rectal cancers: ERUS or MRI?. Surg Oncol Clin N Am.

[49] Taylor FGM, Quirke P, Heald RJ (2011). Pre-operative high-resolution magnetic resonance imaging can identify good prognosis stage I, II and III rectal cancer best managed by surgery alone. A prospective, multicenter, European study. Ann Surg.

[50] Borschitz T, Heintz A, Junginger T (2006). The influence of histopathologic criteria on the long-term prognosis of locally excised pT1 rectal carcinomas: results of local excision (transanal endoscopic microsurgery) and immediate reoperation. Dis Colon Rectum.

[51] Levic K, Bulut O, Hesselfeldt P (2012). The outcome of rectal cancer after early salvage TME following TEM compared with primary TME: a case-matched study. Tech Coloproctol.

[52] O’Neill BD, Brown G, Heald RJ (2007). Non-operative treatment after neoadjuvant chemoradiotherapy for rectal cancer. Lancet Oncol..

[53] Martin ST, Heneghan HM, Winter DC (2012). Systematic review and meta-analysis of outcomes following pathological complete response to neaodjuvant chemoradiotherapy for rectal cancer. Br J Surg.

[54] Hoerske C, Weber K, Goehl J (2010). Long-term outcomes and quality of life after rectal carcinoma surgery. Br J Surg..

[55] Morris E, Quirke P, Thomas JD (2008). Unacceptable variation in abdominoperineal excision rates for rectal cancer: time to intervene?. Gut.

[56] Habr-Gama A, Perez RO, Nadalin W (2004). Operative versus nonoperative treatment for stage 0 distal rectal cancer following chemoradiation therapy: long-term results. Ann Surg.

[57] Habr-Gama A, Perez RO, Proscurshim I (2006). Patterns of failure and survival for nonoperative treatment of stage c0 distal rectal cancer following neoadjuvant chemoradiation therapy. J Gastrointest Surg.

[58] Bedrosian I, Rodriguez-Bigas MA, Feig B (2004). Predicting the node-negative mesorectum after pre-operative chemoradiation for locally advanced rectal carcinoma. J Gastrointest Surg.

[59] Smith FM, Waldron D, Winter DC (2010). Rectum-conserving surgery in the era of chemoradiotherapy. Br J Surg..

[60] Pucciarelli S, Capirci C, Emanuele U (2005). Relationship between pathologic T-stage and nodal metastasis after pre-operative chemoradiotherapy for locally advanced rectal cancer. Ann Surg Oncol.

[61] Bujko K, Nowacki MP, Nasierowska-Guttmejer A (2005). Prediction of mesorectal nodal metastasis after chemoradiation for rectal cancer: Results of a randomised trial: Implication for subsequent local excision. Radiother Oncol.

[62] Mignanelli ED, de Campos-Lobato LF, Stocchi L (2010). Downstaging after chemoradiotherapy for locally advanced rectal cancer: Is there more (tumor) than meets the eye?. Dis Colon Rectum.

[63] Kim DW, Kim DY, Kim TH (2006). Is T classification still correlated with lymph node status after pre-operative chemoradiotherapy for rectal cancer?. Cancer.

[64] Evans J, Patel U, Brown G (2011). Rectal cancer: primary staging and assessment after chemoradiotherapy. Semin Radiat Oncol.

[65] Smith FM, Chang KH, Sheahan K (2012). The surgical significance of residual mucosal abnormalities in rectal cancer following neoadjuvant chemoradiotherapy. Br J Surg.

[66] Borschitz T, Wachtlin D, Möhler M (2008). Neoadjuvant chemoradiation and local excision for T2–3 rectal cancer. Ann Surg Oncol.

[67] Lezoche E, Baldarelli M, Lezoche G (2012). Randomized clinical trial of endoluminal locoregional resection versus laparoscopic total mesorectal excision for T2 rectal cancer after neoadjuvant therapy. Br J Surg.

[68] Garcia-Aguilar J, Shi Q, Thomas CR (2012). A Phase II trial of neoadjuvant chemoradiation and local excision for T2N0 rectal cancer: preliminary results of the ACOSOG Z6041 trial. Ann Surg Oncol.

[69] Allaix ME, Arezzo A, Giraudo G (2012). Transanal endoscopic microsurgery vs laparoscopic total mesorectal excision for T2N0 rectal cancer. J Gastrointest Surg.

[70] Perez RO, Habr-Gama A, São Julião GP (2011). Transanal Endoscopic Microsurgery for residual rectal cancer after neoadjuvant chemoradiation therapy is associated with significant immediate pain and hospital readmission rates. Dis Colon Rectum.

[71] Marks JH, Valsdottir EB, DeNittis A (2009). Transanal endoscopic microsurgery for the treatment of rectal cancer: comparison of wound complication rates with and without neoadjuvant radiation therapy. Surg Endosc.

[72] Bökkerink GMJ, de Graaf EJR, Punt CJA (2011). The CARTS study: chemoradiation therapy for rectal cancer in the distal rectum followed by organ-sparing transanal endoscopic microsurgery. BMC Surgery.

[73] Auyang ED, Hungness ES, VAziri K (2009). Natural orifice translumenal endoscopic surgery (NOTES): dissection for the critical view of safety during transcolonic cholecystectomy. Surg Endosc.

[74] Fong DG, Ryou M, Pai RD (2007). Transcolonic ventral wall hernia mesh fixation in a porcine model. Endoscopy.

[75] Ryou M, Fong DG, Pai RD (2007). Dual-port distal pancreatectomy using a prototype endoscope and endoscopic stapler: a natural orifice transluminal endoscopic surgery (NOTES) survival study in a porcine model. Endoscopy.

[76] Sylla P (2010). Current experience and future directions of completely NOTES colorectal resection. World J Gastrointest Surg.

[77] Sylla P, Rattner DW, Delgado S (2010). NOTES transanal rectal cancer resection using transanal endoscopic microsurgery and laparoscopic assistance. Surg Endosc.

[78] Morino M, Allaix ME, Famiglietti F (2013). Does peritoneal perforation affect short- and long-term outcomes after transanal endoscopic microsurgery?. Surg Endosc.

[79] Salm R, Lampe H, Bustos A (1994). Experience with TEM in Germany. Endosc Surg Allied Technol.

[80] Baatrup G, Borschitz T, Cunningham C (2009). Perforation into the peritoneal cavity during transanal endoscopic microsurgery for rectal cancer is not associated with major complications or oncological compromise. Surg Endosc.

